# Pith parenchyma plasmoptysis: another tissue tension phenomenon driven by osmotic water fluxes

**DOI:** 10.7717/peerj.21493

**Published:** 2026-06-26

**Authors:** Winfried S. Peters

**Affiliations:** 1 Senckenberg Research Institute and Natural History Museum Frankfurt, Senckenberg—Leibniz Institution for Biodiversity and Earth System Research, Frankfurt am Main, Germany; 2 Department of Biological Sciences, Purdue University Fort Wayne, Fort Wayne, IN, United States of America

**Keywords:** Cell wall, Elastic cell wall strain, Elongation growth, Growth zone, Hypocotyl, Plant biomechanics, *Ricinus communis* L., Tissue tension, Turgor pressure

## Abstract

Tissue tension phenomena, rapid changes in size and/or shape of plant organ segments upon their isolation, commonly are interpreted as indicators of biomechanic conditions that control growth and morphogenesis in the intact organ. Studying tissue tension in homogeneous pith parenchyma isolated from etiolated Castor bean (*Ricinus communis* L.) hypocotyls, I found that excisates actually shrank upon isolation, showing ‘spontaneous’ expansion, the classical tissue tension response of isolated inner tissues, only when brought into contact with an exogenous water source. Pith parenchyma segments also were able to expand by recruiting water from other tissues if they remained hydraulically connected. Surprisingly, rapid water-induced elongation to >120% of the original length was followed by shrinkage to below the initial size. This unexpected response depended on external osmolarity, and was caused by plasmoptysis—cell wall rupture—due to excessive osmotic water uptake. While the isolation of pith parenchyma from other tissues is a necessary condition for these effects to occur, none of them can be explained as an elastic response to the release of compressive tissue stresses acting on the pith in intact organs. Instead, the essential role of water fluxes in the causation of tissue tension phenomena is confirmed.

## Introduction: osmotic cells

Biological cells are osmotic cells. Due to the semipermeability of their plasma membranes, biological cells exposed to hypotonic external media take up water osmotically, which increases intracellular hydrostatic pressure (Pfeffer, 1877; Philip, 1958; Nobel, 2020). In prokaryotes, fungi, algae, bryophytes, and vascular plants, this turgor pressure may be substantial as it is contained mechanically by external cell walls, which consequently experience tensile stress and expand elastically (Peters, Hagemann & Tomos, 2000). Turgor pressure enables cell growth, the irreversible cell enlargement against the mechanical resistance of the walls (Geitmann & Ortega, 2009; Cosgrove, 2022), because elastic cell wall strain may develop into plastic, *i.e.,* permanent deformations of the cell wall through incompletely understood molecular processes (Zhang et al., 2021; Chen et al., 2025). The walls, on the other hand, control morphogenesis as they yield differentially to tensile stresses in three dimensions, thanks to their mechanical anisotropy (Baskin, 2005; Baskin & Jensen, 2013). Cells regulate cell wall mechanical anisotropies through complex mechanisms of wall synthesis, assembly, and loosening (Boyer, 2009; Szymanski & Cosgrove, 2009; Robinson et al., 2013; Braidwood, Breuer & Sugimoto, 2014; Coen & Cosgrove, 2023). When these regulatory processes malfunction, or when turgor rises abnormally, wall stresses may exceed the walls’ tensile strength. Overstressed walls may rupture, ejecting a part of the living protoplasm as they rapidly contract to their unstressed dimensions. Historically, studies of such catastrophic cell wall failure and shrinkage—or *plasmoptysis* (“expectoration of protoplasm”), a term introduced by Fischer in 1900—promoted the understanding of biological cells as osmotic systems (Küster, 1929).

In multicellular plants, developmental biomechanics is complicated—or simplified, depending on the research question asked—by the symplastic nature of primary organ growth. Walls of neighboring cells are linked and act as mechanical units (Atakhani, Bogdziewiez & Verger, 2021). Consequently, the positions of cells relative to each other remain fixed in the growth process, and the three-dimensional cell wall network deforms as a whole, *i.e.,* symplastically (Priestley, 1930; Sinnott & Bloch, 1939; intrusive growth of certain cell types may occur at later developmental stages, Lev-Yadun, 2001). Because differential rates of wall loosening across symplastic arrays of cells cannot be balanced by cell movements, they create gradients of reversible extension, or elastic cell wall strain (*ɛ*) across the arrays (Pfeffer, 1893; Heinich, 1909; Thimann & Schneider, 1938). These gradients become manifest as so-called tissue tension phenomena when tissue layers are isolated or when organ symmetry is disturbed, *e.g.*, by cutting a growing internode lengthwise (Peters & Tomos, 1996a). In these cases, organ or tissue segments undergo changes in size and/or shape that are significantly faster than growth *in situ*. If evaluated carefully, tissue tension phenomena may provide clues to the mechanics of the intact organ (Boudaoud, 2010), although the interpretation is not always straightforward and unambiguous (Vincent & Jeronimidis, 1991; Peters & Tomos, 1996a; Peters & Tomos, 1996b; Peters, 2009; Thompson, 2009; Silveira et al., 2025).

A common tissue tension phenomenon in growing stems is observed when the outer tissue consisting of epidermis and, if present, subepidermal collenchyma is peeled off the organ. Outer tissue peels rapidly shrink, while the isolated inner tissues expand, at least when incubated in water. According to classical studies, the experiment works analogously when peripheral portions of isolated inner tissues are removed from more central parts. When isolated from a growing dicot internode, the pith parenchyma (the central, innermost tissue) will elongate more than the vascular tissue, which in turn will become longer than the more peripheral cortex parenchyma, and the epidermis will be shortest (Sachs, 1874 *p. 767*; for an English translation, see Sachs, 1875 *p. 717*). This type of experiment has been conducted routinely with internodes of many species, in student labs as well as original research. Reported time-courses of the expansion of isolated inner tissues or parts thereof are always similar. Peeled hypocotyl segments placed in water, for instance, elongate rapidly but at quickly declining rate; elongation levels off after a few tens of minutes, when the tissue has reached up to 1.4 times its original length (for an example, see Peters & Tomos, 2000).

Castor bean (*Ricinus communis* L.) had been analyzed in seminal works on dicot seedling anatomy (Chick, 1899) and later became a model system for studies into growth mechanics (Meshcheryakov, Steudle & Komor, 1992; Eisenbarth & Weig, 2005) and long-distance transport (Köckenberger et al., 1997; Kalusche et al., 1999). Because its large etiolated hypocotyl lends itself to dissection by inexperienced students, I evaluated its suitability for classroom demonstrations of tissue tension phenomena. Surprisingly, isolated pith parenchyma kept in tap water first expanded as expected but soon shrank back to below its original size. Here I provide a quantitative analysis of this previously unreported tissue tension phenomenon and discuss its implications for our understanding of the mechanics of plant growth and morphogenesis.

## Materials & Methods

### Plant material and *in situ* growth

**Figure 1:**
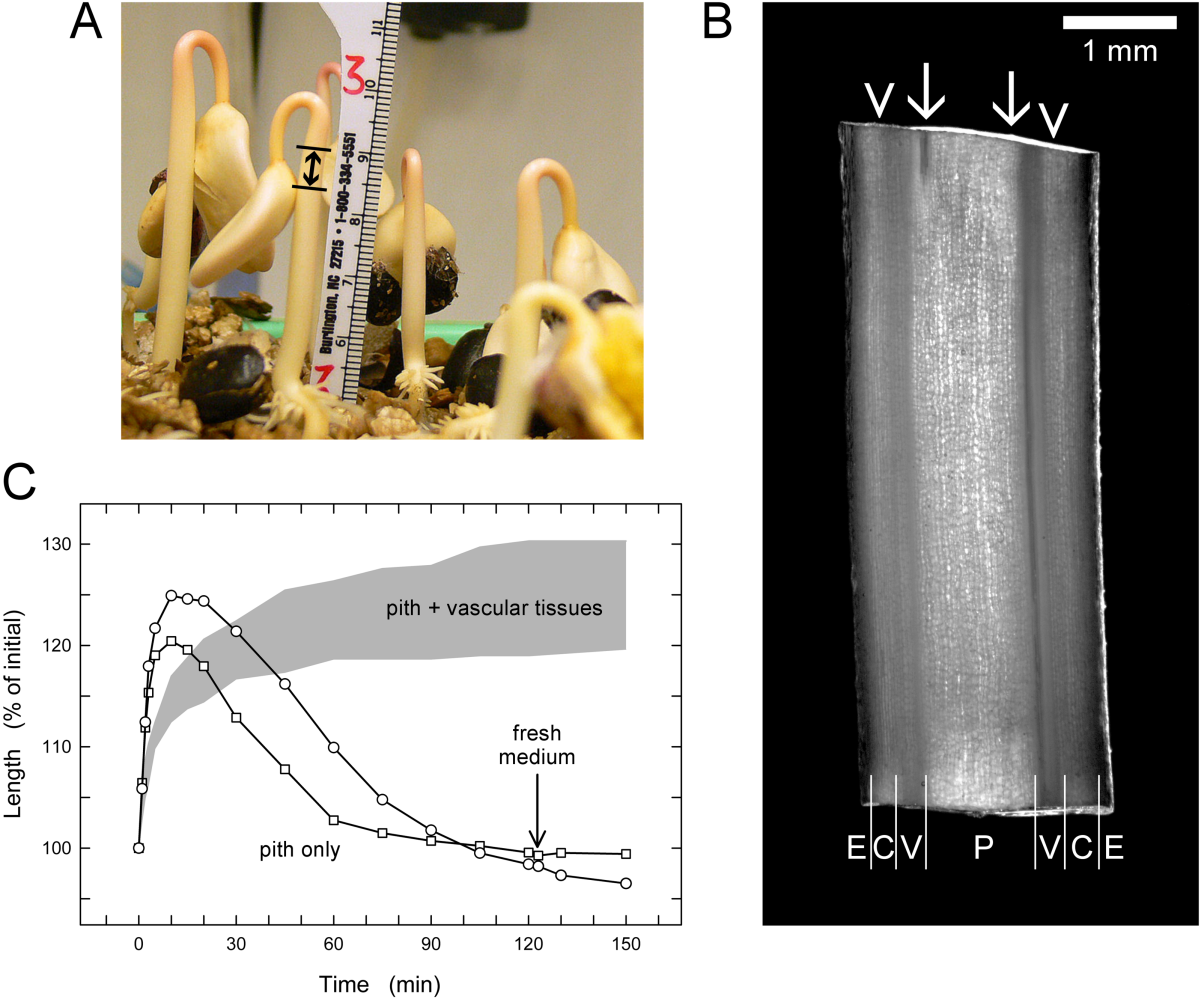
Structure and behavior of inner tissues from the growth zone of etiolated Castor bean hypocotyls. (A) Etiolated seedlings; the position from which 6-mm segments were excised for experimentation is indicated in the specimen to the left of the ruler. (B) Central lamella excised from a hypocotyl; major tissues are indicated at the bottom (E, epidermis; C, cortex parenchyma; V, vasculature; P, pith parenchyma). Arrows and arrowheads on top indicate the cuts made to isolate pith parenchyma or pith parenchyma with vascular tissues, respectively. (C) Representative class-room experiment. Excisates consisting of pith parenchyma plus vascular tissues (four replicates) and of pith parenchyma only (two replicates) were incubated in 10 mL tap water each immediately after excision at time 0. While the pith-plus-vasculature segments expanded as expected (the area covering the trajectories of all four excisates shows in gray), the expansion of the pith segments without vascular tissues (circles) was transient and followed by tissue shrinkage. After 120 min, pith parenchyma segments were transferred to fresh tap water as indicated, without response.

Castor bean (*Ricinus communis* L.; Bay Farm Services, Bay City MI, USA) seeds were soaked in running tap water for 20 h, sown on moist vermiculite, and kept in a dark growth cabinet at 27 °C until the etiolating hypocotyls measured between 4.5 and 5.5 cm from the uppermost lateral root to the apex of the hook (compare Fig. 1A). To quantify elongation of the central growth zone *in situ*, ink marks were made on the hypocotyl epidermis at 0.5 and 1.5 cm below the hook apex. The plants were returned to the growth cabinet; 1 and 4 h later, the marked segments were photographed (digital camera DSC-H20, Sony, Tokyo, Japan) with a ruler for reference. Then, the plants were kept in the dark for another day to verify that the light exposures required for marking and photography (three periods, <90 s each) had been insufficient to induce photomorphogenesis. The lengths of the marked segment on the first and second photograph (*L*_0_ and *L*_t_, respectively) were determined with ImageJ (http://rsbweb.nih.gov/ij/). The relative rate of change, *r*, of a parameter *L* is defined as (Radford, 1967; Peters & Bernstein, 1997):

(1) \begin{eqnarray*}r=dL~d{t}^{-1}~{L}^{-1}.\end{eqnarray*}

Therefore, the relative elongation rate of hypocotyl segments averaged over the period between two measurements (Δ*t*; here, the 3 h that passed between the two photographs), resolves to

(2) \begin{eqnarray*}r= \left( \ln \nolimits ~{L}_{\mathrm{t}}-\ln \nolimits ~{L}_{0} \right) ~\Delta {t}^{-1}.\end{eqnarray*}

### Tissue preparation and observation

The separation of inner tissues from outer tissues occurred in the last step of the preparation, so that the observation of isolated inner tissue could start when it still had its original shape and size. Columns of pith parenchyma were prepared by cutting the apical part of the seedling shoot off at five mm below the apex of the hook. The apical end of the remaining hypocotyl stump was sectioned by two longitudinal cuts of 10 mm length; the two outer segments were removed. The remaining central lamella represented a thick (0.8–1.2 mm), longitudinal stem section (Fig. 1B), from which a segment of six mm length was excised with a custom-made cutter. The pith parenchyma was isolated under a stereomicroscope by two cuts on the inner sides of the vascular bundles (segments consisting of pith parenchyma plus vasculature were made by cutting on the outer sides of the vascular bundles; Fig. 1B). The entire procedure took place in air; with some practice, it was completed in 30–40 s. For *source tissue*-experiments, similar pith parenchyma columns were prepared that remained connected at their proximal (lower) ends to intact hypocotyl segments of 10 or 22 mm length. Prepared tissues immediately were placed in a test medium, either potassium phosphate buffer (15 mOsm KH_2_PO_4_/K_2_HPO_4_, pH 6.3, osmotic pressure *π* ≈ 0.04 MPa; MES, PIPES, and NaH_2_PO_4_/Na_2_HPO_4_ were used as alternative buffer substances in control tests), water-saturated paraffin oil (White Heavy Mineral Oil; Mallinckrodt Baker, Phillipsburg NJ, USA), or aqueous solutions with osmotic pressures (*π*) between 0.2 and 0.8 MPa (potassium phosphate buffer plus appropriate amounts of mannitol; *π* was checked with a 4130 Vapor Pressure Osmometer, Wescor Inc., Logan UT, USA). Time-lapse photograph series of tissue segments were recorded using a Z16 APO macroscope with a DFC490 digital camera controlled by the IM1000 software (Leica Microsystems, Wetzlar, Germany) at resolutions of 6 µm per pixel or better, depending on magnification. The time that passed from the final isolation of the pith parenchyma until the first photograph was taken was ≤3 s. This first photograph defined time 0 for subsequent analyses. Segment lengths were measured on images with ImageJ and segmental *r* was calculated as above (Eq. (2)).

### Turgor-induced elastic cell wall strain and growth analysis

To abolish turgor, plasmolysis was induced by transferring pith parenchyma segments to 2 M NaCl; plasmolyzed segments were photographed after 30 min (control tests had shown that plasmolytic shrinkage was complete after 15 min). Turgor-induced elastic cell wall extension was quantified as the ratio of the length change under stress and the unstressed length, known as engineering or Cauchy strain, *ɛ*; it was calculated from a segment’s turgescent and plasmolyzed length (*L*_tg_ and *L*_pl_, respectively):

(3) \begin{eqnarray*}= \left( {L}_{\mathrm{tg}}-{L}_{\mathrm{pl}} \right) {L}_{\mathrm{pl}}^{-1}.\end{eqnarray*}

To establish continuous time-courses of turgescent and plasmolyzed segment length, of their relative growth rates, and of elastic cell wall strain, segments were kept in potassium phosphate buffer for defined periods before plasmolysis. A highly versatile function for describing skewed single-peak distributions, the five-parameter Weibull function included in the non-linear curve-fitting library of SigmaPlot v.11.0 (Systat Software Inc., San Diego CA, USA), was fitted to the time-course data of turgescent segment length as well as to that of elastic strain:

(4) \begin{eqnarray*}y=\mathrm{a}+~\mathrm{b}{\mathrm{G}}^{ \frac{1-\mathrm{c}}{\mathrm{c}} }  \left\vert { \left( \frac{x-\mathrm{d}}{\mathrm{e}} +{\mathrm{G}}^{ \frac{1}{\mathrm{c}} } \right) }^{\mathrm{c}-1} \right\vert ex{p}^{ \left( - \left\vert { \left( \frac{x-\mathrm{d}}{\mathrm{e}} +{\mathrm{G}}^{ \frac{1}{\mathrm{c}} } \right) }^{c} \right\vert ~+\mathrm{G} \right) }\end{eqnarray*}

where G = (c –1) c^−1^. The time-course of plasmolyzed segment length was described adequately by the much simpler

(5) \begin{eqnarray*}y=\mathrm{a} \left( 1-ex{p}^{\mathrm{b}x} \right) .\end{eqnarray*}

Time-courses of the relative growth rates of turgescent and plasmolyzed segment length were determined using the time derivatives of the fitted functions according to Eq. (1).

### Statistics

Student’s *t*-test and ANOVA with Tukey’s HSD test were conducted at http://vassarstats.net/. Regression functions were fitted by the standard least-square method (SigmaPlot v.11.0, Systat Software Inc., San Diego CA, USA). All sample sizes, *n*, represent biological replicates.

## Results

### Initial observations

Short sections (∼6 mm length; Figs. 1A, 1B) of pith parenchyma isolated from the apical part of etiolating Castor bean hypocotyls of 2 to 12 cm height all showed similar behavior when placed in tap water. They first elongated rapidly to ∼120% of their original length, but then shrank back to their original length or less over 60 to 90 min (Fig. 1C). While the initial elongation was in line with known tissue tension phenomena, the subsequent shrinkage was unexpected. Since the parenchyma cells are packed with starch grains (identified by their characteristic birefringence in the polarization microscope and by iodine/potassium iodide staining) that were released from the cut surfaces of excisates placed in water, it seemed possible that the shrinkage observed was an osmotic response to the accumulation of solutes, specifically glucose, in the external medium. However, transfer of shrinking excisates to fresh tap water had no effect (Fig. 1C). The spontaneous shrinkage of the tissue was evidently not due to an increasingly hypertonic incubation medium, but was part of the tissue’s response to excision and placement in water. This unusual response was specific to homogeneous pith parenchyma, as segments consisting of pith parenchyma with attached vascular tissue exhibited standard behavior—sustained elongation upon placement in water. Since homogeneous pith initially elongated faster than segments with vascular tissues (Fig. 1C), the vasculature seemed to prevent tissue shrinkage by limiting the rate of artificial tissue expansion.

### Growth and turgor-induced cell wall strain *in situ*

**Figure 2:**
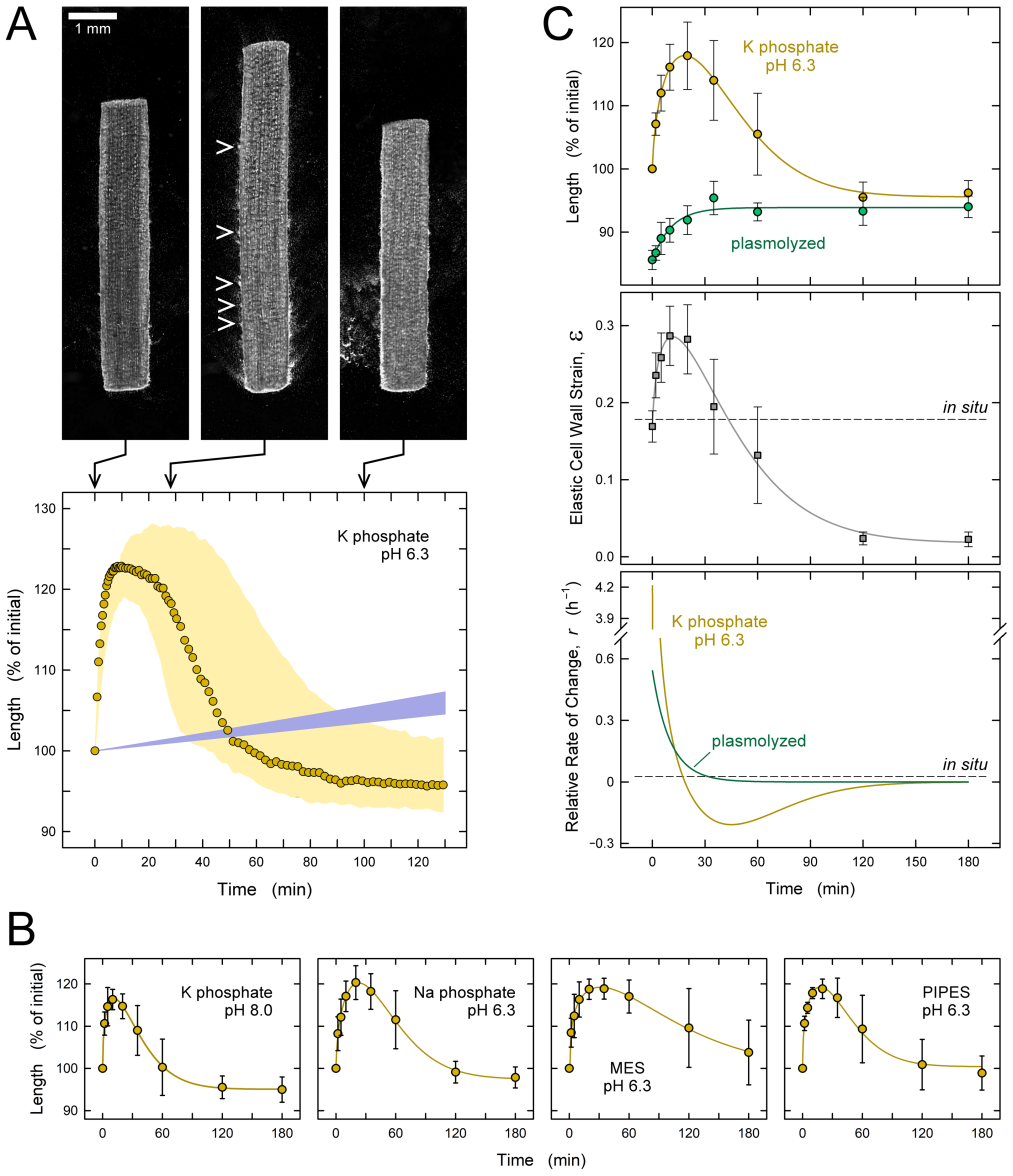
Behavior of isolated pith parenchyma. (A) Micrographs on top show a representative pith parenchyma segment under standard conditions (initial length six mm, incubation in five mL 15 mOsm potassium phosphate buffer, pH 6.3), exhibiting transient expansion and subsequent shrinkage. Arrowheads highlight clouds of starch grains that emerged from distinct points on the segment surface at the time of maximum tissue expansion (compare Video S1). The graph below shows a representative time-course of segment length (circles; tissue preparation completed at time 0); the range covered by time-courses of seven biological replicates is shaded in yellow to visualize variance. For comparison with undisturbed tissue growth, the growth trajectory derived from the mean *in situ* elongation rate ± S.D. is plotted in blue. (B) Similar responses were observed at pH 8 (means ± S.D.; potassium phosphate, *n* = 5) and with other buffers (sodium phosphate, *n* = 5; MES, *n* = 4; PIPES, *n* = 6). (C) Comparison of segment lengths before and after plasmolysis in 2 M NaCl at different stages of the transient expansion and shrinkage responses (top; means ± S.D., *n* = 11 for all datapoints) reveals drastic deviations from the longitudinal elastic strain observed *in situ* (center). Similarly, relative rates of change, *r*, of pre-plasmolyzed and plasmolyzed segment lengths, calculated from regressions fitted to the time-courses of segment length, demonstrate the highly artificial dynamics of the observed deformations.

**Figure 3:**
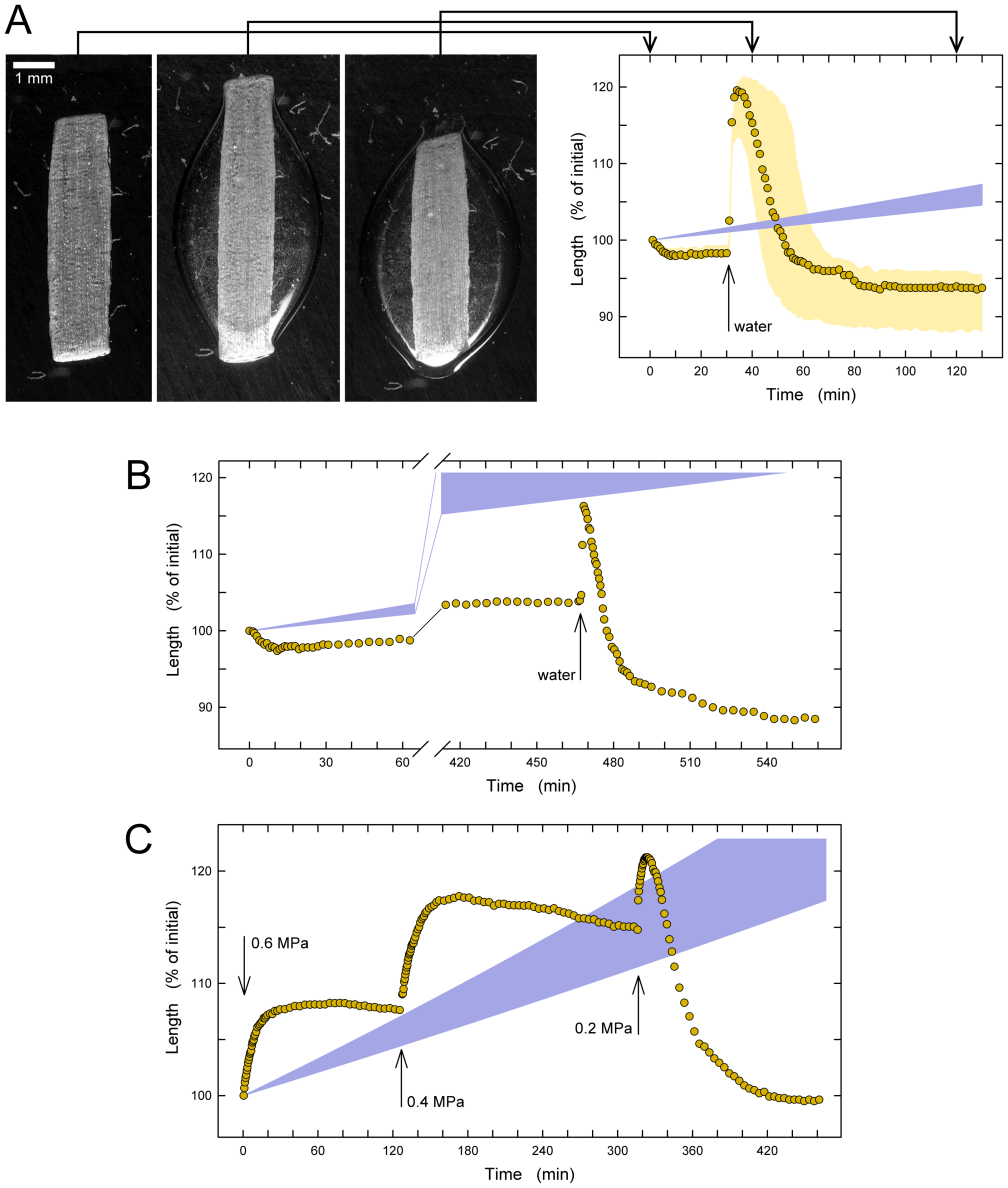
The transient expansion of isolated pith parenchyma requires a water source. (A) In *pith in a bubble*-experiments, parenchyma segments kept under paraffin oil after isolation expanded and shrank when a droplet of water (or standard potassium phosphate buffer, in this case) was placed on the tissue (micrographs on the left; compare Video S2). A representative time-course is shown on the right (circles; tissue preparation was completed at time 0), with the range of data from four biological replicates shaded in yellow. (B) *Pith in a bubble*-experiments can be performed with tissue that had been incubated in paraffin oil for prolonged periods (>7 h in the experiment shown). (C) When external osmotic pressure (*π*) was decreased stepwise (0.6, 0.4, and 0.2 MPa, adjusted with mannitol in standard potassium phosphate buffer), rapid tissue shrinkage occurred only in media of *π* < 0.3 MPa. In all figures, blue wedges represent growth trajectories in the intact plant (mean ± S.D.).

**Figure 4:**
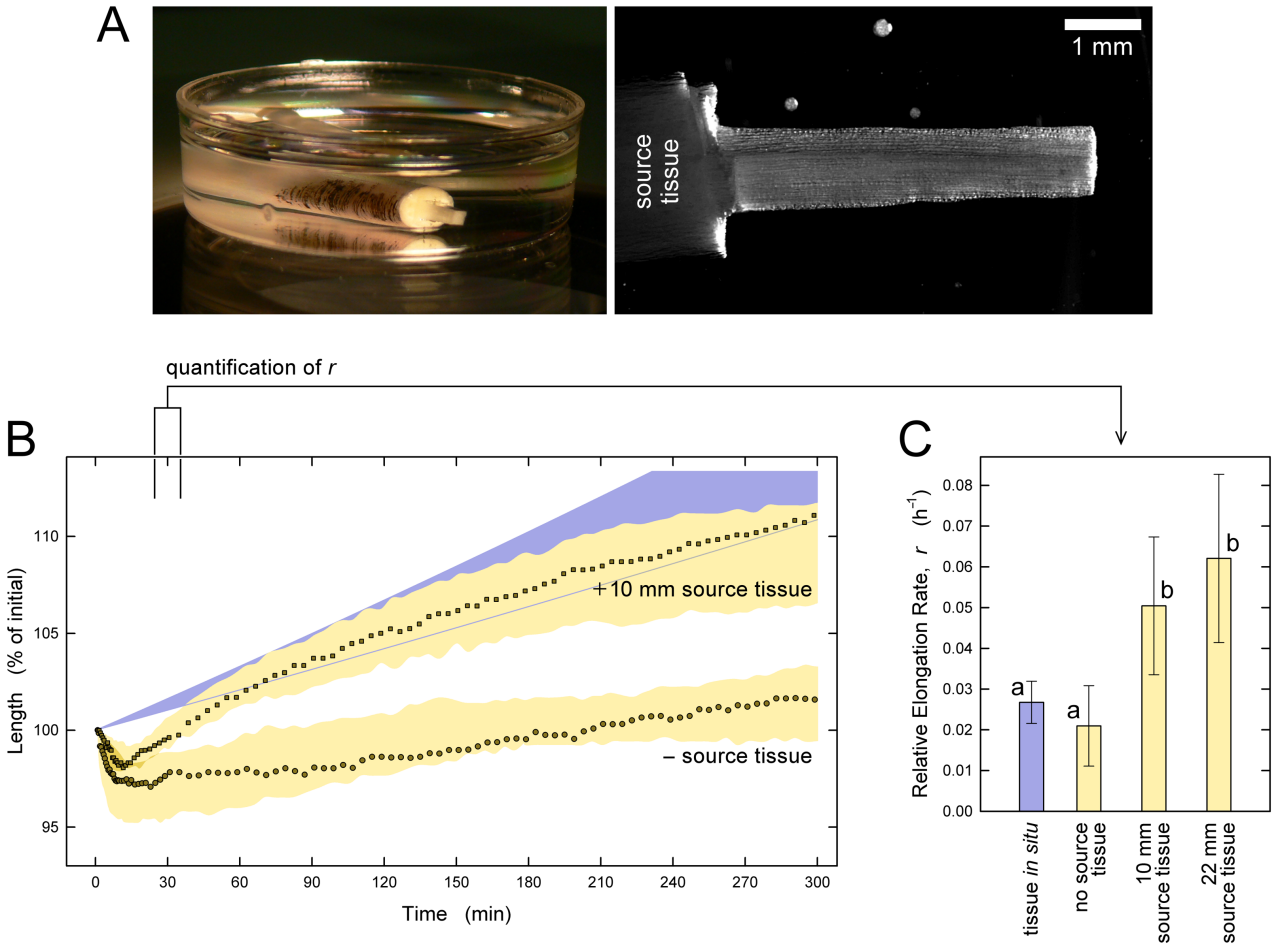
Attached tissues promote the spontaneous expansion of pith parenchyma. (A) A partially isolated pith parenchyma column still connected to an intact hypocotyl segment under paraffin oil, seen in a Petri dish (left) and under the microscope (right); this source tissue may provide water to drive the expansion of the pith column under paraffin oil. (B) Spontaneous elongation of isolated pith parenchyma under paraffin oil, with and without 10 mm source tissue (compare Video S3). Tissue preparation required <30 s and was complete at time 0. For pith columns with and without source tissue, the range of data from eight biological replicates is marked in yellow. One dataset is plotted for each treatment to show within-dataset variability. The blue wedge in the background represents mean *in situ* elongation ± S.D. (C) Relative elongation rates, *r*, of pith parenchyma segments under paraffin oil with 0, 10, and 22 mm source tissue, determined for the period from 25 to 35 min after excision when elongation was most rapid (means ± S.D., *n* = 8 for each of the three treatments). *r* in intact plants (tissue in situ; *n* = 14) corresponds to the blue wedge in (B). Different lower case letters indicate differences at *p* < 0.01 (ANOVA with Tukey’s HSD test).

To elucidate the physical mechanism(s) behind the observed behavior, I characterized it quantitatively using uniform hypocotyls of 4.5 to 5.5 cm height. I first quantified elongation growth *in situ* for the center of the hypocotyl growth zone (0.5 to 1.5 cm below the tip of the hook) from which I intended to excise pith parenchyma segments for subsequent experiments. Over a 3 h period, these one cm segments elongated at a relative elongation rate of 0.027 ± 0.005 h^−1^ (mean ± S.D., *n* = 14). This mean elongation rate *in situ*, *r*_is_, served as the standard to which relative elongation rates under various experimental conditions (*r*_exp_) could be compared. Growth trajectories based on *r*_is_ ± S.D. are shown in Figs. 2A, 3A–3C, and 4B for reference.

To verify that pith parenchyma cell walls were under tensile stress in the growing organ, pith parenchyma segments were plasmolyzed in 2 M NaCl immediately after isolation. Segments shrunk to 84.8 ± 1.9% (mean ± S.D., *n* = 9) of their turgescent length, corresponding to a turgor-induced tensile strain *in situ* of *ɛ*_is_ = 0.179 ± 0.027 in the direction of the organ axis. In other words, turgor elastically extended the longitudinal cell walls *in situ* by 15 to 20% of their unstressed length.

### Pith parenchyma expansion and plasmoptysis

Next, I quantified the transient elongation of excised pith parenchyma observed in the class room (Fig. 1) under standardized conditions. The spontaneous elongation of the excised tissue in potassium phosphate buffer (pH 6.3; osmotic pressure *π* ≈ 0.04 MPa) initially proceeded much faster than growth did *in situ* (Fig. 2; Video S1). Previous studies in sunflower hypocotyls had shown that such rapid elongation of inner tissues following their isolation from peripheral tissues was driven by water uptake and the resulting increases in turgor pressure (Peters & Tomos, 2000). In contrast to sunflower hypocotyl inner tissues, all Castor bean pith excisates had become shorter than the growing tissue would have been *in situ* by 2 h after excision. While starch grains accumulated at comparatively low densities around the tissue immediately after its placement in the medium, distinct plumes of grains began to develop over well-defined spots on the tissue surface at the time shrinkage set in (Fig. 2A). Inspection at higher magnification revealed that these plumes resulted from the rapid release of cell contents (Video S1). The observed tissue shrinkage seemed unlikely to be an artefact caused by the chemical nature of the standard incubation medium (potassium phosphate buffer, pH 6.3), since similar results were obtained with tap water (Fig. 1C), three different buffers at pH 6.3 and potassium phosphate buffer at pH 8.0 (Fig. 2B).

Plasmoptysis results in the abolishment of turgor pressure, and thus in the release of turgor-induced elastic tensile strain in the cell walls. Therefore, if shrinkage were caused by plasmoptysis as the above observations suggested, elastic cell wall strain in the shrinking tissue should decline to values close to zero, and in any case much lower than strain is in the undisturbed, turgescent plant. To test this prediction, I subjected distinct batches of pith segments to the same experimental conditions as above (Fig. 2A), but abolished turgor by plasmolyzing them at different times after preparation. Comparison of segment lengths just before and after completed plasmolysis (Fig. 2C, top) allowed to determine longitudinal, turgor-induced elastic cell wall strain at the time after tissue isolation at which plasmolysis was started (Fig. 2C, center; plasmolyzed segments were discarded once their lengths had been measured). Initially, the length of the plasmolyzed, turgorless tissue increased more slowly but roughly in parallel with the rapid elongation of the turgescent tissue (Fig. 2C, top). However, when the length of the turgescent tissue went past its peak at about 20 min after preparation, plasmolyzed length reached a plateau at about 10% above its initial level. The length of the shrinking, non-plasmolyzed tissue approached this plateau from above by 120 min (Fig. 2C, top). Consequently, elastic cell wall strain reached a level of *ɛ* = 0.023 ± 0.009 (mean ± S.D., *n* = 22) at 180 min (Fig. 2C, center), a mere 13% of its value *in situ* (*ɛ*_is_ = 0.179; the two-tailed Student’s *t*-test yields *p* < 0.0001 for this difference). This finding supported the conclusion that tissue shrinkage was due to plasmoptysis. Because the turgor-induced, tensile elastic cell wall strain increased by 70% over the first 10 min of the experiment, mechanical cell wall failure was not entirely unexpected.

The rapid increase of plasmolyzed segment length over the first 20 min of the experiments demonstrated that irreversible, *i.e.,* plastic deformation of the cell walls occurred as soon as the experiment started. During this period, relative elongation rates of plasmolyzed and non-plasmolyzed segment length were orders of magnitude larger than the relative elongation rate of the tissue growing *in situ* (Fig. 2C, bottom), indicating a highly artificial mechanical state of the expanding cell walls. This observation corroborated the previous conclusion that biomechanical parameters determined in isolated, expanding inner tissues are not necessarily valid estimates of these parameters in undisturbed organs (Peters & Tomos, 2000), which adds to the complications in analyzing the inhomogeneity of tissue mechanical properties *in situ*.

### Rapid parenchyma expansion and plasmoptysis are driven by water uptake

The rapid expansion of excised pith parenchyma could be interpreted, first, as the spontaneous reaction of a compressed tissue to the release of the compressive forces exerted by neighbouring tissues, or, second, as the effect of water uptake from the incubation medium, an external water source. The release of forces exerted by the outer on the inner tissues is interpreted as the immediate cause for the observed response in the first case, but only as a necessary condition for the response to occur in the second. To distinguish the two possibilities, I placed pith parenchyma excisates in paraffin oil to deny them access to exogenous water while preventing water loss through evaporation at the same time. In contrast to expectations based on the common notion of inner tissues being in a state of compression, the excised segments did not expand but rather shrank over 10 min to between 96% and 98% of their initial length (Figs. 3A, 3B, and 4B). They then elongated for up to 8 h, the longest period tested, at a rate that was much lower than growth *in situ* (Figs. 3B and 4B). When a droplet of water was placed on top of segments incubated in paraffin oil, elongation at high rate began immediately, followed by shrinkage to below the initial segment length (*pith in a bubble*-experiments: Figs. 3A, 3B; Video S2). This response was consistently found regardless of the time the tissue had spent under paraffin (>7 h in Fig. 3B), demonstrating that the incubation in oil as such had no significant effects on the osmotic properties of the tissue for the duration of the experiments. Evidently, excised pith parenchyma performed the sequence of rapid expansion and subsequent shrinkage as soon as access to exogenous water was provided, rather than as a direct response to the liberation from mechanical forces exerted by peripheral tissues. In line with this conclusion, pith parenchyma excisates behaved like osmometers when exposed to media of decreasing osmolarities. Osmotic pressures (*π*) below 0.8 MPa induced elongation in a *π*-dependent manner, but only media of *π* < 0.3 MPa triggered the sequence of expansion and shrinkage (Fig. 3C).

### Water re-distribution between tissues can drive parenchyma expansion

I had observed two growth responses; a rapid one (*r*_exp_ >> *r*_is_) requiring water uptake from an external source (Figs. 3A, 3B), and a slow one (*r*_exp_ < *r*_is_) that occurred in the absence of exogenous water (Fig. 3B, before the addition of water). Julius Sachs had explained similarly slow responses in other experimental systems by the re-distribution of water from shrinking cells to expanding ones (Sachs, 1874 *pp. 774–776*, English: Sachs, 1875 *pp. 724–726*). To see whether such a mechanism generally was possible in hypocotyls, I prepared pith parenchyma columns that remained connected at their proximal (lower) ends to intact hypocotyl segments of 10 or 22 mm length (Fig. 4A). These intact segments could potentially serve as water sources for the partially isolated pith parenchyma. Under paraffin oil, pith segments connected to source tissue initially contracted similarly but less strongly as segments without source tissue did (Fig. 4B). After ∼15 min, segments with source tissue started to expand, soon reaching the length that they would have established in the intact plant at the same time (Fig. 4B; compare Video S3). Segments with source tissue elongated significantly faster than the corresponding tissue *in situ* (*r*_exp_ > *r*_is_) at 25 to 35 min after preparation, the time when *r*_exp_ peaked, and segments with 22 mm source tissue showed slightly higher *r*_exp_ than those with 10 mm source tissue (Fig. 4C). In contrast, segments without source tissue elongated slowly with a peak *r*_exp_ that was lower than *r*_is_ (Figs. 4B, 4C). If the observed elongation responses were elastic responses to the removal of assumedly compressing outer tissues, they should be the same with and without source tissues; this is evidently not the case.

## Discussion

### Plasmoptysis

By 1930, plasmoptysis had been described in considerable detail in all major taxa of organisms with walled cells, from prokaryotes to flowering plants (Küster, 1929). Two types of mechanism, which were not mutually exclusive and could occur simultaneously, became recognized (Holdheide, 1931). In *osmotic plasmoptysis*, cell walls literally are blown up, being strained beyond their breaking strength by increasing intracellular pressure due to osmotic water uptake. The effect even is audible when certain marine algae are transferred to freshwater (Úlehla, 1926). Cells progressing towards osmotic plasmoptysis expand as the increasing turgor strains the walls ever more strongly. Upon wall rupture, however, turgor drops to zero and the walls contract. Alternatively, plasmoptysis may be triggered at constant turgor by modifications of cell wall structure that decrease its mechanical strength. Such *defect plasmoptysis* can be induced in root hairs, pollen tubes, and fungal hyphae by exposure to weakly acid media (Klemm, 1895 *pp. 658–663*; Lopriore, 1895).

Importantly, plasmoptysis is not only evoked under artificial experimental conditions. Discharges of cellular contents in *physiological plasmoptysis* have essential functions in reproduction, as in the release of sperm nuclei from pollen tubes (Dresselhaus, Sprunck & Wessel, 2016) and the ejection of fungal spores from asci (Read & Beckett, 1996), or in inter-species interactions such as the establishment of endosymbiosis between the fungus *Geosiphon* and the cyanobacterium *Nostoc* (Mollenhauer, Mollenhauer & Kluge, 1996). Today, these tightly regulated processes are not usually referred to as plasmoptysis anymore, and their link to cellular osmoregulation rarely is addressed in the current literature; the most recent comprehensive treatment is Küster (1958).

The shrinkage of pith parenchyma reported here carries the hallmarks of osmotic plasmoptysis. Tissue segments elongated at a manifold of the natural growth rate (Fig. 2C, bottom), and elastic cell wall strain—the reversible stretch experienced by the walls—rose drastically (Fig. 2C, center) before the extrusion of intracellular material (starch grains) occurred (Fig. 2A). All this happened only if an external water source was available (Figs. 3A, 3B), and depended on the osmolarity of that source (Fig. 3C). Evidently, the cell walls of the pith parenchyma were unable to restrict osmotic water influx once the pith was separated from more sturdy peripheral tissues (Fig. 1C). The resulting burst in turgor strained the walls beyond their limit (this postulated turgor burst actually was measured in inner tissue excisates from sunflower hypocotyls under similar experimental conditions; see Fig. 6 in Peters & Tomos, 2000). Cell wall rupture caused turgor loss and elastic wall strain dropped to near zero (Fig. 2C, center). This did not seem to happen in all cells at the same time, as the resulting tissue shrinkage was protracted over one hour or more (Fig. 2).

In the late 19th century and into the period of plasmoptysis studies, research into the developmental biology of plants generally had a strong biomechanical focus, and tissue tension phenomena received great interest. I am not aware, though, of any previous reports of plasmoptysis occurring in tissue tension experiments.

### Pith plasmoptysis and the developmental-hydraulic interpretation of tissue tension

The biophysical concepts that frame the modern view of the plant cell protoplast as a pressurized osmotic system that grows by stressing its external wall to the point of plastic yielding were not established before the late 19th century (de Vries, 1877; Pfeffer, 1877). Around 1900, it had become common knowledge among botanists that differential rates of “wall growth” in a symplastically growing organ lead to mechanical states in which cells whose wall growth lags behind become load-bearing for the entire organ, limiting the actual growth rate also of those cells that loosen their walls more rapidly (for an exemplary text, see Heinich, 1909, and its discussion by Peters, 2009). Consequently, given a specific turgor pressure, the walls of most cells do not expand elastically as they would in the isolated cell, but either less or more than that, depending on the cell’s rate of wall growth relative to that of other cells in the symplastic organ. In the undisturbed plant, gradients of wall mechanical properties are not necessarily apparent because they are balanced; intact growing organs exist in dynamic mechanical equilibria. If cell layers are separated experimentally, however, the balance is disturbed and new mechanical equilibria become established. Tissue tension phenomena—the bending, shrinkage, or unusually rapid expansion of excisates and organ parts—represent visible manifestations of the establishment of such new equilibria. In addition to the internal re-distribution of water between the tissues and cell layers, the availability of exogenous water has a strong influence on these new mechanical equilibria, since living cells are osmotic systems. It seemed a trivial insight that an inner tissue cell, which *in situ* was prevented from further expansion by rigid outer tissues, would not be able to expand upon isolation from the outer tissues unless it could take up water to fuel its expansion. I refer to this time-honored and empirically well-supported interpretation as the *developmental-hydraulic model of tissue tension* (Peters, 2009). How do the above results fit into the picture?

The developmental-hydraulic model predicts that if isolated homogenous pith parenchyma cannot acquire water from other, connected tissues, it will expand rapidly only if an exogenous water source is available. This is the case (Figs. 3A, 3B). Since cells take up water by osmosis, the model further predicts that the degree of excisate expansion will depend on the osmolarity of the external water source. This is the case as well (Fig. 3C). The model as expressed by Sachs (1874) implies that the innermost tissue, if freed from the mechanical constraints of outer tissues but still connected hydraulically to these tissues, will expand by water re-distribution from the outer tissues. My results accord with this prediction (Fig. 4). Last but not least, the peculiar delayed shrinkage of excised pith parenchyma due to plasmoptysis following excessive water uptake and dramatic increases of elastic cell wall strain is compatible with although not necessarily predicted by the model, which in the form described above makes no assumptions concerning the tensile strength of cell walls. Taken together, the present findings integrate effortlessly into the developmental-hydraulic model.

### Significance of water fluxes

Today, it seems to be a widely accepted principle of plant biomechanics that “inner tissues, composed of larger cells, are generally under compression, while the epidermis is under tension” (Lapointe et al., 2025 *p. 7*), which seems to imply that “internal tissue expands when the epidermal constraint is removed” (Coen & Cosgrove, 2023 *p. 5*). However, in the above experiments, the removal of outer tissues from the pith did not trigger expansion; the contact of the isolated pith with water did (Figs. 3A, 3B), confirming previous observations in isolated inner tissues (Heinich, 1909; Hejnowicz & Sievers, 1995; Peters & Tomos, 2000). Evidently, the removal of outer tissues establishes a necessary condition for the rapid expansion of the pith, but does not cause its expansion, just like the release of the parking brake as such does not accelerate an automobile. Since causal analyses of physical processes require the distinction of necessary conditions from immediate causes, I stress that the immediate cause of the expansion of inner tissue excisates is water uptake. This fact has been disputed (Kutschera & Niklas, 2007) although plant cells and tissues generally need to take up water from their environment to swell or grow (Forterre, 2013).

The consideration of water fluxes and a clear distinction between elastic (*i.e.,* reversible) and plastic (*i.e.,* irreversible) cell wall deformations (as documented in Fig. 2C) are essential for a consistent interpretation of tissue tension phenomena in the context of organ biomechanics and growth (Heinich, 1909; Peters & Tomos, 2000; Peters, 2009). The monitoring of silicon oil-incubated pith parenchyma that could neither lose water by evaporation nor draw water from external sources, commenced within 3 s after the separation from outer tissues was completed. Rather than expanding instantaneously as one would expect from a compressed tissue, the pith excisates contracted (Figs. 3A, 3B, 4B; see also Hejnowicz & Sievers, 1995). Assuming that tissue tension phenomena represent direct responses to the release of physical stresses between cell layers in the intact plant (Kutschera & Niklas, 2007; Lapointe et al., 2025), one would conclude that prior to isolation, the pith was held in a state of tension—not compression—by the outer tissues. An explanation more closely aligned with physical principles governing the behavior of osmotic cells is that cell sap, liberated by the cuts that isolated the pith, increased the osmolarity of the aqueous solution in the cell walls of the excisate, thus withdrawing water from the living cells, which consequently shrank.

In the biomechanics of turgescent plant cells, turgor pressure is not a constant, but a highly dynamic parameter. If a turgescent plant cell experiences moderate changes in turgor or volume, new mechanical equilibria will be established by appropriate water fluxes across the plasma membrane (Hüsken, Steudle & Zimmermann, 1978). The time required for completion of these fluxes generally is short; in cells of growing hypocotyls, it has been found to be in the range of seconds (Meshcheryakov, Steudle & Komor, 1992; Peters & Tomos, 2000; Eisenbarth & Weig, 2005). It follows that compared to the time needed for tissue preparation in most published tissue tension experiments, water redistributes almost instantaneously in response to changes in water potential gradients on the cellular level. This questions the validity of conclusions regarding intact organ biomechanics from measurements of mechanical parameters performed on excisates some time after preparation. The capacity for rapid water redistribution on the cellular level translates into water fluxes over larger distances that occur over commensurably longer periods (Steudle, 1997; Steudle & Peterson, 1997; Wegner & Zimmermann, 2008). Such fluxes can drive growth (Sachs, 1874 *p. 776*, English: Sachs, 1875 *pp. 725–726*; Matyssek, Tang & Boyer, 1991) and plant movements (Irving et al., 1997; Dumais & Forterre, 2012; Forterre, 2013) through the mere re-distribution of water within organs; Fig. 4 provides an additional example. Skotheim & Mahadevan (2005) suggested that water flux-driven plant movements including tissue swelling and shrinkage can be distinguished from elastic effects by the relationship between the duration of the movement, *τ*, and the smallest macroscopic dimension of the moving part, *L*. A critical value of 1.6 s/mm^2^ separates movements limited by fluid transport (*τ*/*L* ^2^ > 1.6 s/mm^2^) from movements that rely on elastic instabilities (*τ*/*L* ^2^ < 1.6 s/mm^2^). With a maximum estimate of 1.5 mm for the diameter of the isolated pith parenchyma segments studied here, I arrive at a maximum period of 3.6 s for the completion of purely elastic processes. The durations of all pith parenchyma deformations presented above exceed this value by at least two orders of magnitude, demonstrating that these deformations cannot be explained as elastic responses to the release of forces that may act between tissue layers in the intact plant. Rather, the conclusion is corroborated that the observed deformations reflect water fluxes, in agreement with the developmental-hydraulic interpretation of tissue tension.

## Conclusion: Back to the Roots

In the early 20th century, seemingly intuitive mechanical metaphors began to replace the developmental-hydraulic interpretation of tissue tension. As a popular textbook explained in 1923, neither “a weak spiral spring …made [of] thin steel wire” nor a segment of “an inner tube of a cycle-tyre …is capable of supporting itself in an erect position; if, however, the spring is slipped into the rubber tube and the former slightly compressed, so that the ends of the tube can be firmly tied, the two combined form a structure of considerable rigidity. Here, just as in the stem, the inner part is in a state of compression while the outer is extended” (Fritch & Salisbury, 1923 *p. 104*). By representing growing plant organs as a combination of elastic yet solid elements rather than as what they really are—complex osmotic cells—the *spring in a tube*-analogy and similar metaphors turn a necessary condition for the expansion of isolated inner tissues into the expansion’s immediate cause—after all, a compressed spring will in fact expand as soon as all outer constraints are removed. In reality, however, all turgescent plant tissues including the pith consist of two components of which one, the cell wall network, is under tension, while the other, the living protoplasm, is under compression. Inner tissues are no structural analogues of compressed spiral springs, and since they evidently do not expand like compressed springs do when isolated, they are no mechanical analogues either. But if one accepts the analogy as valid, it will appear self-evident that inner tissues must expand immediately when freed from the constraints of outer tissue layers; that this expansion is independent of water fluxes; and that expansion of the entire system—*i.e.,* organ growth—is controlled mechanically by the outer tissues (see, for example, Kutschera & Niklas, 2007). Simple, easily reproducible experiments like the ones reported here contradict such interpretation. I suggest that recent steps towards a generally applicable hydromechanical field theory of plant morphogenesis (Oliveiri & Cheddadi, 2025) will gain additional momentum if we take the developmental-hydraulic views favored by our colleagues of the 19th century seriously again.

##  Supplemental Information

10.7717/peerj.21493/supp-1 Supplemental Information 1 Transient expansion of isolated pith parenchyma (A) Spontaneous transient expansion. (B) Starch grain release from distinct points on the tissue surface. (C) Release of starch grains from a single cell.

10.7717/peerj.21493/supp-2 Supplemental Information 2 ”Pith in a bubble” experiment The response of a pith parenchyma segment kept under paraffin oil to the placement of a droplet of water on its surface.

10.7717/peerj.21493/supp-3 Supplemental Information 3 “Source tissue” experiment The continuous expansion of a pith parenchyma segment still connected to a complete (i.e., including all tissues in their natural arrangement) hypocotyl segment.

10.7717/peerj.21493/supp-4 Supplemental Information 4 Raw Data

## Additional Information and Declarations

### Data Availability

The following information was supplied regarding data availability: The data is available in the Supplemental Files.
